# Encephalitis in HIV-negative immunodeficient patients: a prospective multicentre study, France, 2016 to 2019

**DOI:** 10.2807/1560-7917.ES.2024.29.6.2300046

**Published:** 2024-02-08

**Authors:** Sophie Landré, Florence Ader, Olivier Epaulard, Pierre Tattevin, Jean Paul Stahl, Alexandra Mailles, on behalf of the Steering committee and Investigators

**Affiliations:** 1 University Claude Bernard Lyon 1, Hospices Civils de Lyon, Infectious disease department, Lyon, France; 2 Centre International de Recherche en Infectiologie (CIRI), Inserm 1111, Université Claude Bernard Lyon 1, CNRS, UMR5308, École Normale Supérieure de Lyon, Univ Lyon, France; 3 ESCMID Study Group on the infections of the Brain (ESGIB), Basel, Switzerland; 4 University Grenoble Alpes, Infectious diseases department, Grenoble, France; 5 Infectious diseases department, CHU Pontchaillou, Rennes, France; 6 Santé Publique France, Saint-Maurice, France; 7 The members of the Scientific committee and the investigators are listed in the Acknowledgments section

**Keywords:** encephalitis, immunodeficient patient, cohort

## Abstract

**Background:**

Data on infectious encephalitis in immunodeficient (ID) individuals are scarce. This population may present with atypical clinical symptoms, be infected by uncommon pathogens and develop poor outcomes.

**Aim:**

We aimed to describe the epidemiology of infectious encephalitis among HIV-negative ID patients.

**Methods:**

Patients from the ENCEIF (Etude Nationale de Cohorte des Encéphalites Infectieuses en France) prospective cohort meeting criteria for infectious encephalitis between January 2016 and December 2019 were included. We compared clinical presentation, magnetic resonance imaging (MRI) results, biological results, infection causes and outcome of ID patients with immunocompetent (IC) patients using Pearson’s chi-squared test and Student’s t-test. We carried out logistic regression to assess the role of immunodeficiency as risk factor for poor outcome.

**Results:**

ID patients (n = 58) were older (mean 72 vs 59 years), had higher prevalence of diabetes (26% vs 12%), pre-existing neurological disorders (12% vs 5%) and higher case-fatality rate (23.6% vs 5.6%) compared to IC patients (n = 436). Varicella zoster virus was the primary cause of encephalitis in ID patients (this aetiology was more frequent in ID (25.9%) than in IC patients (11.5%)), with herpes simplex virus second (22.4% in ID patients vs 27.3% in IC patients). Immunodeficiency was an independent risk factor for death or major sequelae (odds ratio: 3.41, 95%CI: 1.70–6.85).

**Conclusions:**

Varicella zoster virus is the most frequent cause of infectious encephalitis in ID patients. Immunodeficiency is a major risk factor for poor outcome. ID encephalitis patients should benefit from stringent investigation of cause and early empiric treatment.

**Key public health message**

**What did you want to address in this study?**

We wanted to investigate whether infectious encephalitis among patients with a weak immune system (immunodeficient patients) for reasons other than HIV, had particularities with regards to clinical presentation, microbiological findings, magnetic resonance imaging (MRI) results and outcome. We compared these data between immunodeficient and immunocompetent (non-affected immune system) French patients between 2016 and 2019.

**What have we learnt from this study?**

Encephalitis in both groups of patients is quite similar in terms of clinical presentation and MRI findings. However, the varicella zoster virus was the primary cause of encephalitis in immunodeficient patients, while the herpes simplex virus was the primary cause in immunocompetent patients. Immunodeficiency was an independent risk factor for death or severe sequelae.

**What are the implications of your findings for public health?**

Immunodeficient and immunocompetent patients should benefit from a stringent investigation of the cause of their encephalitis and early antiviral and antibiotic empiric treatment. Physicians should be aware of the severity of this disease among immunodeficient patients.

## Introduction

Infectious encephalitis is a rare disease with an estimated incidence of 7.3 per 100,000 persons per year in the United States (US) between 2000 and 2010 [1]. It has a high case-fatality rate (CFR) (around 10%) and neurological sequelae are frequent; 3 years after hospital discharge, 32% of patients presented severe or moderate sequelae [2,3]. Encephalitis can be challenging to diagnose considering the clinical overlap with other neurological conditions. The cause of encephalitis can be even more difficult to determine due to the variety of possible involved pathogens [4,5].

The life expectancy of immunodeficient (ID) populations is increasing as new indications for immunosuppressive treatments are validated and management of onco-hematological disorders or transplantations improves, leading to increased life expectancy and risk of complications from infections. In central nervous system infections, ID patients may display atypical clinical presentation, and since they are sensitive to infections that usually do not affect immunocompetent patients (opportunistic infections), they may develop a more severe outcome [6].

Previous studies on encephalitis in ID patients mostly included patients with acquired immunodeficiency syndrome due to human immunodeficiency virus (HIV). To date, to the best of our knowledge, no survey of a prospective cohort of encephalitis in HIV-negative ID patients has been reported.

Our objective was to evaluate differences in infectious encephalitis between non-HIV-related ID patients and immunocompetent (IC) patients enrolled in the ENCEIF cohort (Etude Nationale de Cohorte des Encéphalites Infectieuses en France), a prospective multicentre cohort of infectious encephalitis in France [7]. We included clinical presentation, the causative agent of encephalitis, biological results, magnetic resonance imaging (MRI) results, treatment and outcome.

## Methods

### Enrolment of patients

The ENCEIF Cohort has previously been described elsewhere [7,8]. Patients with suspected infectious encephalitis matching the stringent case definition adapted from Venkatesan et al. [9] and admitted to one of the 62 hospital units throughout metropolitan France between 1 January 2016 and 31 December 2019 were included in this study. Investigators were encouraged to apply the French Infectious Diseases Society guidelines when investigating encephalitis aetiology [10].

### Data collection

Investigators collected demographic data, as well as data on comorbidities, ongoing treatment, clinical presentation (fever > 38.0 °C, confusion, coma, septic shock, hallucination), encephalitis causative agent, biological results, MRI findings described by the local radiologist, treatment and outcome. Glasgow outcome scale (GOS) was used to assess the global functioning of patients at discharge: a poor outcome was defined as a GOS of 1 to 3 (death, neurovegetative state or major sequelae) [11]. We defined immunodeficiency as having: (i) an active cancer; (ii) ongoing chemotherapy within the past year; (iii) undergone a solid organ or haematopoietic stem cell transplantation; (iv) a haematological malignancy; (v) a myelodysplastic syndrome; or (vi) undergoing immunosuppressive treatment.

### Statistical analysis

The variables collected for both groups of patients were described using medians and interquartile range (IQR) for quantitative variables and numbers and percentage for qualitative variables. In the univariate analysis, variables were compared using Pearson’s chi-squared test and Student’s t-test (using odd ratios (OR) and 95% confidence intervals (CI)). Risk factors for poor outcome were assessed with multivariable analysis using a logistic regression model. All variables associated with a poor outcome in the univariate analysis with a p value < 0.20 were included in an initial multivariable logistic regression model. The variable selection was performed according to a backward-stepwise procedure. The causative agent and immunodeficiency were retained in the model irrespective of the significance of their association with the dependent variable (encephalitis outcome). The goodness-of-fit of the final model was evaluated by the Hosmer-Lemeshow test [12].

Data were computed using Voozanoo version 3 (Epiconcept, Paris, France) and analysed using Stata statistical software, version 16.0 (Stata Corp, College Station, US).

## Results

### Patient characteristics

The ENCEIF cohort enrolled 494 patients, of whom 58 (11.7%) matched the criteria for ID patients (Table 1). The median age of ID patients was 72 years old (IQR: 68–78), 37 (64%) were male and 21 (36%) were female. Compared with IC patients (n = 436), ID patients were older (mean 72 years vs 59 years, p < 0.001), were more prone to diabetes (26% (15/58) vs 12% (52/433), p = 0.004), and had more pre-existing neurological disorders (12% (7/58) vs 5% (22/433), p = 0.003). Sex ratio and distribution of educational background levels were similar.

**Table 1:** Characteristics of immunosuppressive disorders in immunodeficient HIV-negative infectious encephalitis patients ENCEIF cohort, France 2016–2019 (n = 58)

Immunosuppressive disorder	Patients		Details
	n	%	
Immunosuppressive treatment for an auto-immune disease^a^	19	32.8	• Corticosteroids alone (n = 11) • Corticosteroids combined with other immunosuppressive agents (rituximab and methotrexate, two patients each) (n = 4) • Immunosuppressive agents without corticosteroids (methotrexate, adalimumab plus methotrexate, adalimumab, and non-specified TNF inhibitor) (n = 4)
Haematological malignancy	15	25.9	• Chronic lymphocytic leukaemia (three patients receiving treatment) (n = 6) • Myeloma (all patients receiving treatment including two on revlimid and one autologous haematopoietic stem cell transplant) (n = 3) • Lymphoma: non-Hodgkin lymphoma and Hodgkin’s lymphoma (two patients with recent chemotherapy including rituximab, nivolumab, and revlimid) (n = 3) • Waldenström disease (one patient with recent chemotherapy) (n = 2)
Transplantation	15	25.9	• Heart transplant^b^ (n = 3) • Kidney transplant^b^ (n = 7) • Liver transplant^b^ (n = 1) • Allogeneic haematopoietic stem cell transplant^b^ (n = 4)
Active cancer or recent chemotherapy (< 1 year)	8	13.8	• Prostate cancer (one patient undergoing chemotherapy) (n = 2) • Glioblastoma (patient undergoing chemotherapy) (n = 1) • Melanoma (patient undergoing chemotherapy and taking nivolumab) (n = 1) • Bladder cancer (no chemotherapy) (n = 1) • Pulmonary cancer (patient undergoing chemotherapy) (n = 1) • Colon cancer (patient undergoing chemotherapy) (n = 1) • Breast cancer (patient undergoing chemotherapy) (n = 1)
Myelodysplasia	2	3.4	• Myelofibrosis (n = 1) • Myelodysplastic syndrome (n = 1)

ENCEIF: Etude Nationale de Cohorte des Encéphalites Infectieuses en France; TNF: tumour necrosis factor.

^a^ Systemic disease: rheumatoid arthritis, polymyalgia rheumatica, giant cell arteritis, polymyositis, Schulmann fasciitis, granulomatosis with polyangiitis.

^b^ All patients received immunosuppressive treatment except one recipient of hematopoietic stem cell allograft.

One patient had immunodeficiency of multiple origin.

### Encephalitis causative agents

Varicella zoster virus (VZV) was the primary cause of encephalitis in ID patients (25.9% vs 11.5% in IC patients, p = 0.002) (Table 2). Herpes simplex virus (HSV) was the second most common cause in ID patients (22.4%) but did not significantly differ to IC patients (27.3%). *Listeria monocytogenes* was the third most common cause of infectious encephalitis in ID patients (10.3% in ID vs 3.9% in IC, p = 0.03). Human herpes virus 6 (HHV-6) and John Cunningham (JC) polyomavirus infections were identified in ID patients only. The proportion of cases without an infectious agent identified was lower in ID patients than in IC patients (12.1% vs 37.4%, p = 0.0001).

**Table 2:** Causes of infectious encephalitis in HIV-negative immunodeficient and immunocompetent patients, ENCEIF cohort, France 2016–2019 (n = 494)

Causative agent	Immunodeficient patients N = 58		Immunocompetent patients N = 436		p value
	n	%	n	%	
VZV	15	25.9	50	11.5	0.002
HSV	13	22.4	119	27.3	0.43
*Listeria monocytogenes*	6	10.3	17	3.9	0.029
*Mycobacterium tuberculosis*	0	0	11	2.5	0.22
TBE virus	2	3.4	24	5.5	0.51
Influenza virus	0	0	11	2.5	0.22
EBV	2	3.4	4	0.9	0.10
CMV	0	0	1	0.2	0.71
HHV6	2	3.4	0	0	10^− 4^
JC virus	3	5.2	0	0	< 10^− 4^
*Cryptococcus neoformans*	3	5.2	1	0.2	< 10^− 4^
Other causes^a^	5	17.2	35	8.5	NA
Unknown	7	12.1	163	37.4	10^− 4^

CMV = cytomegalovirus; EBV = Epstein-Barr virus; ENCEIF: Etude Nationale de Cohorte des Encéphalites Infectieuses en France; HHV6 = human herpes virus 6; HSV = herpes simplex virus; JC virus: John Cunningham virus; NA: not applicable; TBE = tick borne encephalitis; VZV = varicella zoster virus.

^a ^Other causative agents: among immunocompetent patients: enterovirus (n=5), *Mycoplasma pneumoniae* (n = 4), measles virus (n = 3), West Nile virus (n = 3), *Capnocytophaga canimorsus* (n = 2), *Leptospira* spp. (n = 2), Japanese encephalitis virus (n = 2), *Tropheryma whipplei* (n = 2), *Anaplasma phagocytophilum* (n = 1), Chikungunya virus (n = 1), *Coxiella burnetii* (n = 1), *Legionella pneumophila* (n = 1), *Borrelia burgdorferi* (n = 1), Human parvovirus B19 (n = 1), *Treponema pallidum* (n = 1), Toscana virus (n = 1), *Francisella tularensis* (n = 1), HIV (n = 1), Zika virus (n = 1), *Rickettsia* spp. (n = 1).

Other causative agents among immunodeficient patients: *Borrelia burgdorferi* (n = 2), West Nile virus (n = 1), *Bartonella* spp. (n = 1), enterovirus (n = 1).

### Clinical data

Clinical presentation, including frequency of fever at admission, did not differ between ID and IC patients (Table 3).

**Table 3:** Clinical symptoms at admission, in HIV-negative patients, ENCEIF cohort, France 2016–2019 (n = 494)

Clinical symptoms	Immunodeficient patients N = 58		Immunocompetent patients N = 436		p value
	n	%	n	%	
Fever	41	73.2	354	81.6	0.14
Confusion	40	71.4	325	77.2	0.34
Altered consciousness	37	63.8	250	58.4	0.43
Coma	11	20.0	78	18.5	0.79
Septic shock	3	5.2	21	4.8	0.91

ENCEIF: Etude Nationale de Cohorte des Encéphalites Infectieuses en France.

Magnetic resonance imaging was performed equally often in both groups (81.2%, 354 /436 IC patients and 79.3%, 46 /58 ID patients). However, due to missing data, MRI results of only 34 ID patients and 230 IC patients were included in this study. The prevalence of haemorrhagic lesions was higher in ID patients (23.5%, 8/34) than IC patients (12.6%, 29/230, but this difference did not reach statistical significance (p = 0.09). No specific clinical presentation or MRI findings were associated with a specific cause of infection in ID patients.

The distribution of white blood cell (WBC) count/mm^3^ in cerebrospinal fluid (CSF) was significantly different in ID and IC patients (p = 0.03). A median WBC count of 45/mm^3^ (IQR: 6–183) was reported for ID patients, and a median WBC count of 80/mm^3^ (IQR: 22–268) for IC patients. The absence of biological meningitis, defined as a CSF WBC count < 5/ mm^3^, was more common in ID patients (23.6%, 13/55) than in IC patients (8.9%, 38/423) (p = 0.005).

After excluding patients with early diagnoses through direct microbiological examination of their CSF, namely patients with *L. monocytogenes* and tuberculosis, acyclovir was less frequently prescribed to ID patients (76.0%, 38/50) than IC patients (92.7%, 371/401), (p = 0.0001). Of note, all patients with a final diagnosis of encephalitis caused by HSV or VZV had received acyclovir as empirical treatment. The proportion of patients who received a high dose of acyclovir (15 mg/kg/8 hours) was similar in both groups (24.6%, 90/366 IC patients vs 36.1%, 13/36 ID patients, p = 0.13).

### Outcome

In-hospital CFR was significantly higher in ID patients (23.6%) than in IC patients (5.6%), p < 0.001. Causes of infectious encephalitis in non-survivor ID patients were: VZV (n = 4), HSV (n = 2), JC virus (n = 2), *L. monocytogenes* (n = 1), Epstein-Barr virus (n = 1), and *Cryptococcus neoformans* (n = 1), no causative agent was identified for two ID patients. Major sequelae at discharge were more frequent in ID patients (20%) than in IC patients (94%), p = 0.001 (Table 4).

**Table 4:** Functional outcome at hospital discharge in HIV-negative immunodeficient and immunocompetent patients with infectious encephalitis, ENCEIF cohort, France 2016–2019 (n = 482)

Outcome	Immunodeficient patients N = 55		Immunocompetent patients N = 427		p value
	n	%	n	%	
Death	13	23.6	24	5.6	< 10 ^− 3^
Neuro-vegetative state	0	0	2	0.5	0.65
Major sequelae	11	20	40	9.4	10 ^− 3^
Moderate sequelae	10	18.2	110	25.7	0.63
Minor sequelae	21	38.2	251	58.8	0.12

ENCEIF: Etude Nationale de Cohorte des Encéphalites Infectieuses en France.

Functional outcome is assessed with the Glasgow outcome scale [11].

Outcome data were not available for three immunodeficient patients and nine immunocompetent patients.

In the final multivariable model, non-HIV-related immunodeficiency, age, coma and abnormal MRI findings were independently associated with poor outcome (Table 5). Having no causative agent identified was independently associated with a good outcome. The model goodness-of-fit was 0.84.

**Table 5:** Risk factors for poor outcome from infectious encephalitis in HIV-negative immunodeficient patients from the multivariable analysis, final model, ENCEIF cohort, France 2016–2019 (n = 465)

Variable	OR	95% CI	p value
Age^a^	1.04	1.02–1.05	< 10^− 3^
Immunodeficiency	3.41	1.70–6.85	10^− 3^
Coma	6.05	3.31–11.07	< 10^− 3^
MRI lesions	3.47	1.86–6.65	< 10^− 3^
No causative agent identified	0.47	0.21–0.82	0.012

CI: confidence interval; ENCEIF: Etude Nationale de Cohorte des Encéphalites Infectieuses en France; GOS: Glasgow outcome scale; MRI: magnetic resonance imaging; OR: odds ratio.

Immunocompetent patients were the reference group for OR calculations. ORs were adjusted for immunodeficient patients.

^a^ For every 1-year increase in age.

Poor outcome was defined as a GOS of 1 to 3 (death, neurovegetative state or major sequelae).

## Discussion

The main findings of this multicentre, prospective cohort study on infectious encephalitis in HIV-negative ID patients are as follows: (i) ID patients with infectious encephalitis were older and had a higher prevalence of diabetes and pre-existing neurological disorders than IC patients; (ii) VZV was the primary cause of infectious encephalitis in ID patients; (iii) poor outcome was 2.7 times more frequent in ID patients (41.4%) than in IC patients (15.4%); (iv) having an immunodeficiency is an independent risk factor for death or major sequelae in HIV-negative patients with infectious encephalitis. The older age of ID patients with encephalitis was expected, since the prevalence of ID conditions increase with age. However, ID patients, representing 11.7% of the ENCEIF cohort, had a similar clinical presentation to IC patients.

The distribution of infectious causes of encephalitis differed between the two groups. Among ID patients, the most frequent causative agent was VZV (26%), followed by HSV (22%), as opposed to IC patients, wherein HSV was the most frequent cause identified (27%) and VZV the second (11%).

This finding might suggest increasing the dose of acyclovir from 10 mg/kg/8 hours to 15 mg/kg/8 hours for the empirical treatment of encephalitis in HIV-negative ID patients before identifying the causative agent. However, although VZV is less susceptible to acyclovir than HSV in vitro [13], there are no data showing that a higher dose is more effective than a lower one, and there has been no clinical trial so far. Moreover, weighing the benefits and risks advocates against the systematic use of high-dose acyclovir for empirical treatment of encephalitis, given the dose-dependent renal and neurological toxicity of acyclovir [14]. Hence, most international guidelines recommend a dose of 10 mg/kg/8 hours acyclovir for empirical treatment of encephalitis, except when the probability of VZV encephalitis is high [10,15].

Our finding that VZV is the most frequent cause of encephalitis in HIV-negative ID patients has consequences: (i) the criteria for the use of high-dose acyclovir in this population may be different than for IC patients (i.e. consider high-dose in the presence of any suggestive clinical or imaging findings as haemorrhagic lesions) [16-18]; (ii) the results of CSF PCR tests for both HSV and VZV should be obtained as soon as possible in ID encephalitis patients to adjust the antiviral treatment if needed [10].

The apparent lower rate of acyclovir prescription in ID patients in this study raises concerns, given that HSV and VZV remain the most frequent pathogens responsible for encephalitis in this population, and given the strong association between early treatment with acyclovir and outcome [10,15]. However, none of the patients in this study with an apparent absence of acyclovir were later diagnosed with HSV and VZV.

As expected, *L. monocytogenes* was significantly more frequent among ID patients than IC patients.

Other opportunistic pathogens associated with severe immunodeficiency were found in our study, namely HHV-6, JC polyomavirus, and *C. neoformans*. Human herpes virus 6 may be responsible for encephalitis only in ID patients [19], and was identified in one allogeneic haematopoietic stem cell transplant recipient and one patient receiving long-term corticosteroid treatment in our cohort. Human herpes virus 6 integration was ruled out in those two cases. We diagnosed three cases of JC polyomavirus encephalitis: two patients with lymphoma treated with chemotherapy, and one heart transplant recipient who recently received rituximab treatment [20]. The two cases of Epstein-Barr virus encephalitis occurred in kidney transplant recipients in our cohort.

Encephalitis of unknown cause was three times less frequent among ID patients (12.1%) than IC patients (37.4%). Although the ENCEIF cohort only enrolled patients with encephalitis presumably of infectious origin, some cases of unknown origin may have been immune or inflammatory encephalitis. In HIV-negative ID patients with a weak immune system due to immunosuppressive treatment and unable to exhibit a strong immune or inflammatory response, encephalitis of inflammatory cause may be less frequent. This hypothesis is supported by our finding that having an encephalitis of unknown cause despite the suspicion of infectious origin was an independent protective factor against poor outcome in our final multivariable model. It is also possible that ID patients were more comprehensively investigated than IC patients. However, our data do not allow us to assess this hypothesis.

Several non-infectious causes of central nervous system disorders may be more common in HIV-negative ID patients, such as posterior reversible encephalopathy syndrome in patients treated with calcineurin inhibitors [21], paraneoplasic encephalitis [22], central nervous system inflammation caused by tumour necrosis factor inhibitors [23] or neurologic adverse effects of immune checkpoint inhibitors [24]. Although non-infectious, these patients may fulfil the ENCEIF inclusion criteria (altered mental status > 24 hours, seizures, confusion, focal neurologic deficit, fever, MRI lesions) and could have been enrolled in our cohort.

The fact that 23.6% of ID patients had no biological meningitis (i.e. CSF WBC count < 5/ mm^3^) outlines that suspected encephalitis should not be ruled out with regards to this finding only, especially in HIV-negative ID patients, who may display a lower inflammatory response. This is in agreement with the international case definition [9], where meningitis is only one criterion among others. In line with our findings, a literature review of encephalitis in ID patients found that HSV and VZV encephalitis were associated with lower CSF WBC count than in IC patients [25].

A retrospective case control study on HSV and VZV encephalitis in HIV-negative ID patients (n = 14), with an ID and IC group comparable to our study patients, showed a lower frequency of prodromal symptoms, an overall similar clinical presentation and a much higher CFR (35.7%) in ID patients than in IC patients (6.7%), in agreement with our study. They also found that a low CSF WBC count was associated with death, which may give way to a physio-pathological hypothesis for the severity of encephalitis in ID patients. Their inability to develop an efficient immune response may enable the pathogen to spread in the brain parenchyma [26].

We documented the high severity of encephalitis among ID patients with in-hospital CFR of 23.6%, and a high prevalence of sequelae or persisting symptoms impairing their global functioning at discharge (20%). Immunodeficiency was an independent risk factor of poor outcome with an OR of 3.41 (95% CI: 1.7–6.85), after adjusting for confounding bias. This finding suggests lowering immunosuppression therapy in HIV-negative ID patients presenting with encephalitis when possible, as a part of encephalitis treatment. An increase in age was also associated with poor outcome, in agreement with a previous study [2,4].

Beside susceptibility to severe infection, a secondary explanation for the severity of HSV or VZV encephalitis among ID patients may be acyclovir resistance due to prolonged exposure to valacyclovir, which is used as the primary prophylaxis in several ID conditions, particularly for transplant recipients [27,28].

Finally, prevention strategies should be used where possible. Varicella zoster virus infections could be avoided through pre-immunodeficiency vaccination, and food-borne listeriosis may be prevented in vulnerable patients, for example by informing them about at-risk food and spreading recommendation about preparing meals and respecting best-before dates [29]. These strategies are critical to address with the patient before starting an immunosuppressive treatment, keeping in mind the need for balance between making drastic changes and taking risk-adapted advice to lower the risk of infectious diseases.

The strengths of our study are the prospective design, allowing extensive data collection, the size of our cohort, a multicentric inclusion of patients and high external validity. To the best of our knowledge, this is the first prospective cohort to present the characteristics of infectious encephalitis among a broad spectrum of immunodepressive conditions.

This study has limitations. First, as this cohort was designed to study the clinical features and risk factors of infectious encephalitis in a large population in France, we did not collect extensive data on immune status, such as dose or duration of corticosteroid use or time elapsed since transplantation. Second, since patients were enrolled by infectious disease specialists, neurologists and intensivists, some patients with severe immunodeficiency may not have been enrolled if they were managed in other departments, such as haematology.

## Conclusion

Immunodeficient patients with infectious encephalitis are at high risk of death or major sequelae. While HIV-negative ID patients have been shown to be similar to IC patients in terms of clinical presentation and MRI results, the prevalence of meningitis was lower in ID patients. Varicella zoster virus, HSV and *L. monocytogenes* were the most frequent causes of infectious encephalitis among ID patients. Although investigating the causative agent should include rare and more ID-oriented pathogens, looking for the common pathogens responsible for infectious encephalitis in IC patients remains essential. Diagnosis and treatment algorithms, with empirical acyclovir, need to be carried out with the same stringency as with IC patients.

## References

[1] George BP Schneider EB Venkatesan A . Encephalitis hospitalization rates and inpatient mortality in the United States, 2000-2010. PLoS One. 2014;9(9):e104169. 10.1371/journal.pone.0104169 25192177 PMC4156306

[2] Mailles A De Broucker T Costanzo P Martinez-Almoyna L Vaillant V Stahl JP Steering Committee and Investigators Group . Long-term outcome of patients presenting with acute infectious encephalitis of various causes in France. Clin Infect Dis. 2012;54(10):1455-64. 10.1093/cid/cis226 22460967

[3] Venkatesan A . Epidemiology and outcomes of acute encephalitis. Curr Opin Neurol. 2015;28(3):277-82. 10.1097/WCO.0000000000000199 25887770

[4] Mailles A Stahl JP Steering Committee and Investigators Group . Infectious encephalitis in france in 2007: a national prospective study. Clin Infect Dis. 2009;49(12):1838-47. 10.1086/648419 19929384

[5] Granerod J Ambrose HE Davies NW Clewley JP Walsh AL Morgan D Causes of encephalitis and differences in their clinical presentations in England: a multicentre, population-based prospective study. Lancet Infect Dis. 2010;10(12):835-44. 10.1016/S1473-3099(10)70222-X 20952256

[6] Castro I, Ruiz J, Tasias M, Montero M, Salavert M. Central nervous system infections in immunocompromised patients. Rev Esp Quimioter. 2018;31(Suppl 1) Suppl 1;56-61. PMC6459576 30209926

[7] Mailles A, Argemi X, Biron C, Fillatre P, De Broucker T, Buzelé R, et al. Changing profile of encephalitis: Results of a 4-year study in France. Infect Dis Now. 2021. 10.1016/j.idnow.2021.11.007 34896660

[8] Le Maréchal M Mailles A Seigneurin A Tattevin P Stahl JP Épaulard O A Prospective Cohort Study to Identify Clinical, Biological, and Imaging Features That Predict the Etiology of Acute Encephalitis. Clin Infect Dis. 2021;73(2):264-70. 10.1093/cid/ciaa598 32433723

[9] Venkatesan A Tunkel AR Bloch KC Lauring AS Sejvar J Bitnun A Case definitions, diagnostic algorithms, and priorities in encephalitis: consensus statement of the international encephalitis consortium. Clin Infect Dis. 2013;57(8):1114-28. 10.1093/cid/cit458 23861361 PMC3783060

[10] Stahl JP Azouvi P Bruneel F De Broucker T Duval X Fantin B Guidelines on the management of infectious encephalitis in adults. Med Mal Infect. 2017;47(3):179-94. 10.1016/j.medmal.2017.01.005 28412044

[11] McMillan T Wilson L Ponsford J Levin H Teasdale G Bond M . The Glasgow Outcome Scale - 40 years of application and refinement. Nat Rev Neurol. 2016;12(8):477-85. 10.1038/nrneurol.2016.89 27418377

[12] Hosmer DW Hosmer T Le Cessie S Lemeshow S . A comparison of goodness-of-fit tests for the logistic regression model. Stat Med. 1997;16(9):965-80. 10.1002/(SICI)1097-0258(19970515)16:9<965::AID-SIM509>3.0.CO;2-O 9160492

[13] Chemaly RF Hill JA Voigt S Peggs KS . In vitro comparison of currently available and investigational antiviral agents against pathogenic human double-stranded DNA viruses: A systematic literature review. Antiviral Res. 2019;163:50-8. 10.1016/j.antiviral.2019.01.008 30677427

[14] Ryan L Heed A Foster J Valappil M Schmid ML Duncan CJA . Acute kidney injury (AKI) associated with intravenous aciclovir in adults: Incidence and risk factors in clinical practice. Int J Infect Dis. 2018;74:97-9. 10.1016/j.ijid.2018.07.002 30048817

[15] Tunkel AR Glaser CA Bloch KC Sejvar JJ Marra CM Roos KL The management of encephalitis: clinical practice guidelines by the Infectious Diseases Society of America. Clin Infect Dis. 2008;47(3):303-27. 10.1086/589747 18582201

[16] Bertrand A Leclercq D Martinez-Almoyna L Girard N Stahl JP De-Broucker T . MR imaging of adult acute infectious encephalitis. Med Mal Infect. 2017;47(3):195-205. 10.1016/j.medmal.2017.01.002 28268128

[17] Le Bot A Ballerie A Pronier C Bénézit F Reizine F Tas M Characteristics and outcome of varicella-zoster virus central nervous system infections in adults. Eur J Clin Microbiol Infect Dis. 2021;40(11):2437-42. 10.1007/s10096-021-04245-y 33907935

[18] Nagel MA Cohrs RJ Mahalingam R Wellish MC Forghani B Schiller A The varicella zoster virus vasculopathies: clinical, CSF, imaging, and virologic features. Neurology. 2008;70(11):853-60. 10.1212/01.wnl.0000304747.38502.e8 18332343 PMC2938740

[19] Seeley WW Marty FM Holmes TM Upchurch K Soiffer RJ Antin JH Post-transplant acute limbic encephalitis: clinical features and relationship to HHV6. Neurology. 2007;69(2):156-65. 10.1212/01.wnl.0000265591.10200.d7 17620548

[20] Hartman EA Huang D . Update on PML: lessons from the HIV uninfected and new insights in pathogenesis and treatment. Curr HIV/AIDS Rep. 2008;5(3):112-9. 10.1007/s11904-008-0018-0 18627659

[21] Song T Rao Z Tan Q Qiu Y Liu J Huang Z Calcineurin Inhibitors Associated Posterior Reversible Encephalopathy Syndrome in Solid Organ Transplantation: Report of 2 Cases and Literature Review. Medicine (Baltimore). 2016;95(14):e3173. 10.1097/MD.0000000000003173 27057842 PMC4998758

[22] Kyritsis AP Markoula S Alexiou G Asimakopoulos A Jabbour P Fotopoulos A Diagnosis and treatment of limbic encephalitis in the cancer patient. Future Oncol. 2020;16(22):1647-55. 10.2217/fon-2020-0080 32511017

[23] Kunchok A Aksamit AJ Jr Davis JM 3rd Kantarci OH Keegan BM Pittock SJ Association Between Tumor Necrosis Factor Inhibitor Exposure and Inflammatory Central Nervous System Events. JAMA Neurol. 2020;77(8):937-46. 10.1001/jamaneurol.2020.1162 32421186 PMC7235930

[24] Larkin J Chmielowski B Lao CD Hodi FS Sharfman W Weber J Neurologic Serious Adverse Events Associated with Nivolumab Plus Ipilimumab or Nivolumab Alone in Advanced Melanoma, Including a Case Series of Encephalitis. Oncologist. 2017;22(6):709-18. 10.1634/theoncologist.2016-0487 28495807 PMC5469590

[25] Saylor D Thakur K Venkatesan A . Acute encephalitis in the immunocompromised individual. Curr Opin Infect Dis. 2015;28(4):330-6. 10.1097/QCO.0000000000000175 26098507

[26] Tan IL McArthur JC Venkatesan A Nath A . Atypical manifestations and poor outcome of herpes simplex encephalitis in the immunocompromised. Neurology. 2012;79(21):2125-32. 10.1212/WNL.0b013e3182752ceb 23136265 PMC3511927

[27] Andrei G Topalis D Fiten P McGuigan C Balzarini J Opdenakker G In vitro-selected drug-resistant varicella-zoster virus mutants in the thymidine kinase and DNA polymerase genes yield novel phenotype-genotype associations and highlight differences between antiherpesvirus drugs. J Virol. 2012;86(5):2641-52. 10.1128/JVI.06620-11 22190713 PMC3302250

[28] Stránská R Schuurman R Nienhuis E Goedegebuure IW Polman M Weel JF Survey of acyclovir-resistant herpes simplex virus in the Netherlands: prevalence and characterization. J Clin Virol. 2005;32(1):7-18. 10.1016/j.jcv.2004.04.002 15572000

[29] Gaudot C . [Prevention of Listeria infections]. Bull Acad Natl Med. 2000;184(2):287-93, discussion 294. 10989538

